# Homozygous deletion of both GSTM1 and GSTT1 genes is associated with higher CD4^+^ T cell counts in Ghanaian HIV patients

**DOI:** 10.1371/journal.pone.0195954

**Published:** 2018-05-24

**Authors:** Joshua Agbemefa Kuleape, Emmanuel Ayitey Tagoe, Peter Puplampu, Evelyn Yayra Bonney, Osbourne Quaye

**Affiliations:** 1 West African Centre for Cell Biology of Infectious Pathogens (WACCBIP), Department of Biochemistry, Cell and Molecular Biology, University of Ghana, Legon, Accra, Ghana; 2 Department of Medicine, Korle Bu Teaching Hospital, Accra, Ghana; 3 Noguchi Memorial Institute for Medical Research, University of Ghana, Legon, Accra, Ghana; Tor Vergata University, ITALY

## Abstract

Glutathione S-transferase (GST) family of enzymes are involved in a two-stage detoxification process of a wide range of environmental toxins, carcinogens and xenobiotics. The GST enzymes play important roles in oxidative stress pathways, and polymorphisms in the GSTM1 and GSTT1 genes mediate susceptibility and outcome in different diseases. Human immunodeficiency virus (HIV) infection is associated with oxidative stress, but there is limited data on the frequency of deleted GSTM1 and GSTT1 genes in HIV/AIDS patients and their effect on progression among Ghanaians. This study sought to investigate the association between homozygous deletion of GSTM1 and GSTT1 genes (both null deletion) with HIV/AIDS disease progression in Ghanaian patients. HIV-infected individuals on antiretroviral therapy (ART), ART-naïve HIV patients, and HIV seronegative individuals were recruited for the study. HIV/AIDS disease progression was assessed by measuring CD4^+^ cell count and viral load of the patients, and GST polymorphism was determined by amplifying the GSTT1 and GSTM1 genes using multiplex PCR, with CYP1A1 gene as an internal control. The mean CD4^+^ count of patients that were naïve to ART (298 ± 243 cells/mm^3^) was significantly lower than that of patients on ART (604 ± 294 cells/mm^3^), and viral load was significantly lower in the ART-experienced group (30379 ± 15073 copies/mm^3^) compared to the ART-naïve group (209882 ± 75045 copies/mm^3^). Frequencies of GSTM1 and GSTT1 deletions were shown to be 21.9% and 19.8%, respectively, in the HIV patients, and patients with homozygous deletion of both GSTM1 and GSTT1 were more likely to have their CD4^+^ count rising above 350 cells/mm^3^ (OR = 6.44, 95% CI = 0.81–51.49, p = 0.039) suggesting that patients with homozygous deletion of GSTM1 and GSTT1 genes have slower disease progression. The findings of this study show that double deletion of glutathione S-transferases M1 and T1 is statistically associated with normal CD4^+^ count in patients diagnosed with HIV/AIDS. Further study is required to investigate the clinical importance of the both null deletion in HIV patients.

## Introduction

Since its discovery more than three decades ago, HIV/AIDS has become one of the leading causes of death worldwide, and an estimated 36.7 million people were reportedly living with HIV as at 2015 [1]. Sub-Saharan Africa is the most affected region and makes up more than 70% of individuals living with HIV worldwide. The incidence of HIV and AIDS related deaths is due to a continuous immunodeficiency associated with the depletion of CD4^+^ T lymphocytes, which leads to a compromised immune system [2].

Glutathione S-transferases (GST) are a family of drug metabolizing enzymes with a high level of conjugation specificity for glutathione (GSH), and the enzymes are essential for metabolism of many substances. There are at least seven groups of enzymes namely alpha (GSTA), mu (GSTM), pi (GSTP), kappa (GSTK), zeta (GSTZ), sigma (GSTS) and theta (GSTT) for detoxification of compounds in drugs and carcinogens, and for inhibition of oxidative damage to tissues [3]. The most studied GST genes are GSTM1 and GSTT1, and they have been described as polymorphic in humans [4]. Polymorphisms in GST are linked to higher risk of oxidative stress, which has been suggested to favour HIV replication [3, 5]. Two groups of polymorphisms have been documented: the homologous deletion genotype (also called the null genotype) and one or two undeleted alleles (called the non-null or present genotype) [4]. Individuals with the null genotype lack the GST enzyme, and studies have suggested that the distribution of the genotypes vary among different ethnic groups [6]. The GSTM1 gene is located on chromosome 1p13.3 and genetic differences in the gene can affect an individual's predisposition to cancer, as well as the toxicity and effectiveness of some drugs [7]. The GSTT1 gene is found on chromosome 22q11.23 and the enzyme is involved in the conjugation and detoxification of drugs containing butadiene epoxide, bromodichloromethane, dichloromethane, ethylene dibromide, methylene chloride and ethylene oxide [8, 9]. The deletion of GSTM1 and GSTT1 genes (both null genotype) has been associated with a rise in a number of cancers, possibly due to an amplified susceptibility to the harmful effects of oxidative stress, environmental toxins and carcinogens [7, 10]. It has been reported that nearly 50% of Caucasians and Asians have the both null genotype [6, 11] but in Nigerians and black South Africans, the frequency is approximately 30% and 20%, respectively [4, 12].

Polymorphisms in GSTM1 and GSTT1 gene sequences affect expression of the GST enzyme while increasing susceptibility to oxidative stress in addition to altered risk of several diseases [13–15]. Furthermore, the GST variants alter the catalytic activity of the enzymes and thus, persons producing less definite detoxification enzymes could be at a higher risk of adverse outcome of disease [16]. Studies that did not observe an association between the null genotype and cancers tried to explain that other members of the GST enzyme family must have compensated for the absence of a functional enzyme in the double deletion subjects [17, 18]. In the current study, the association of GSTM1 and GSTT1 polymorphisms with oxidative stress among Ghanaian HIV/AIDS patients was studied.

## Methods

### Study design and sample collection

This is a cross-sectional study with the study participants recruited from the Fevers Unit of the Korle Bu Teaching Hospital in Accra, Ghana. The study was approved by the Institutional Review Board (IRB) of the Noguchi Memorial Institute for Medical Research, and all the participants signed a consent form after the objectives of the study has been explained to them (Study number 068/14-5). Blood samples were collected from each recruited individual under aseptic conditions into EDTA tubes and used for CD4^+^ count and viral load as well as gene polymorphism analysis. CD4^+^ count was stratified by the Centers for Disease Control and Prevention (CDC) staging criteria [19]. In addition to the CDC stratification, a CD4^+^ count of less than 350 cells/mm^3^ is required for antiretroviral therapy (ART) initiation at the time of the study design. Questionnaires were used to collect data on socio-economic status and medical history of the patients. A total of 242 adults were recruited for the study which included 60 HIV negative individuals as controls, 105 HIV infected patients on ART, and 77 HIV infected patients who are ART-naïve.

### Markers of HIV disease progression

An automated Becton Dickson (BD) FACSCount machine (Becton, Dickson and Company, Brussels, Belgium) was used to determine the absolute count of CD4^+^ T cells in whole blood according to the manufacturer’s instructions. The COBAS AmpliPrep/COBAS TaqMan machine (ROCHE, USA) was used to quantify HIV RNA, as a measure of the viral load, in the plasma samples by following the manufacturer’s protocol.

### GST polymorphism assay

**Fig 1:**
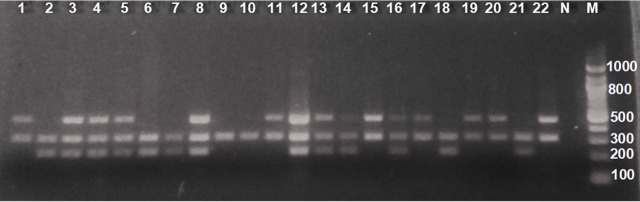
Electrophoresis of PCR products from the co-amplification of GSTM1, GSTT1 and the internal control, CYP1A1 gene. Ethidium bromide stained 2% agarose gel. N = negative control, M = 100bp molecular weight marker. Lanes 2, 6, 7, 18 and 21 were positive for GSTT1 deletion genotype. Lanes 1, 11, 15, 17, 19, 20 and 22 had GSTM1 deletion genotype. Lanes 9 and 10 represented individuals with a deletion of both genotypes. Lanes 3, 4, 5, 8, 12, 13, 14 and 16 had both genes present.

Genomic DNA was extracted from 200 μL of whole blood using the QIAamp DNA Mini kit (Hilden, Germany) per the manufacturer’s protocol, and Nanodrop Lite (Thermo Fischer, Massachusetts, United States) was used to check the purity and yield of the DNA obtained. The DNA was used in a multiplex PCR assay using the Qiagen multiplex PCR kit (Hilden, Germany) with primer sequences and cycling conditions as shown in Gattas *et al*., 2004 [20]. Briefly, 2.5 μL of DNA was amplified in a 50 μL multiplex PCR reaction which contained a mixture of the PCR master mix (32.5 μL) and 2.5 μL each of the following GSTM1 primers (forward– 5’ GAA CTC CCT GAA AAG CTA AAG C 3’ and reverse– 5’ GTT GGG CTC AAA TAT ACG GTG G 3’), GSTT1 primers (forward– 5’ TTC CTT ACT GGT CCT CAC ATC TC 3’ and reverse– 5’ TCA CCG GAT CAT GGC CAG CA 3’), and primers for exon 7 of the CYP1A1 gene (forward– 5' GAA CTG CCA CTT CAG CTG TCT 3' and reverse– 5' CAG CTG CAT TTG GAA GTG CTC 3') which was used as an internal control (Fig 1). The PCR products from the co-amplification of GSTM1 (215 bp) and GSTT1 (480 bp) were visualized on an ethidium bromide-stained 2.0% agarose gel [20]. Based on the bands observed on the gel, the samples were put into two groups as either present or null genotypes.

### GST enzyme activity

GST activity was assayed spectrophotometrically by monitoring the conjugation of 1-chloro-2,4-dinitro benzene (CDNB) with glutathione (GSH) at λ_max_ = 340 nm at 37 ^0^C as previously done [21].

### Statistical analysis

Shapiro-Wilk normality test was used to distinguish between parametric and non-parametric data, with the parametric data represented as mean ± standard deviation. One-way ANOVA was used to analyse differences among the data, followed by a post-hoc analysis. Chi-square test and Fisher Exact test were applied to assess the association between the categorical variables according to pre-established cut-off points. Odds ratios (ORs) and 95% confidence interval (CI) were calculated. IBM SPSS 22 and GraphPad Prism v6 were used for the statistical analysis, and the statistical significance level was set at p-value <0.05.

## Results

The distributions of demographical characteristics are shown in Table 1. Females were more than males, whilst the ART patients were older (45.6 ± 0.9 years p ≤0.01) than the ART-naïve (41.1 ± 1.4 years) and control groups (38.7 ± 1.9 years).

**Table 1:** Basic demographic data of study population stratified by ART use.

	Patient groups		Control (N = 60)	*p*-value
	ART (N = 105)	ART-naïve (N = 77)		
Age (years) ± SE	45.6 ± 0.9	41.1 ± 1.4	38.7 ± 1.9	0.01
Male n (%)	45 (42.9%)	21 (27.3%)	19 (31.7%)	
Female n (%)	60 (57.1%)	56 (72.7%)	41 (68.3%)	

Data are presented as mean ± standard error of mean (SE). *p*-value was obtained from One-way ANOVA. ART = Antiretroviral treatment.

The CD4^+^ count among the individuals on ART (604 ± 294, p ≤0.005) was significantly higher than those who are ART-naïve (298 ± 243) (Table 2). Conversely, viral load among the ART patients (30379 ± 15073, p ≤0.001) was significantly less than those who were not on ART (209882 ± 75045). More than three-quarters of the patients on ART had CD4^+^ counts that were more than 350 cells/mm^3^, whilst 41.5% of the patients who were not on treatment had CD4^+^ counts below 200 cells/mm^3^.

**Table 2:** Comparison of CD4^+^ counts, viral load and CDC staging of CD4^+^ count among the HIV seropositive groups.

	Patient groups		p value
	ART ± SE (N = 105)	ART-naïve ± SE (N = 77)	
CD4^+^ count (cells/mm^3^)	604 ± 294	298 ± 243	0.005*
Viral load (copies/mm^3^)	30379 ± 15073	209882 ± 75045	< 0.001*
CDC staging of CD4^+^ count groups			
< 200 (cells/mm^3^)	5 (4.8%)	32 (41.5%)	<0.001**
200–349 (cells/mm^3^)	17 (16.2%)	16 (20.8%)	
> 350 (cells/mm^3^)	83 (79%)	29 (37.7%)	

Values for CD4^+^ counts and viral load were rounded to the nearest hundredth.

*p values were determined from t-test and

**p value was from the Pearson Chi-square test.

Amplicons from samples positive for GSTM1 and GSTT1 genotypes yielded band sizes of 215 bp and 480 bp, respectively, while the internal positive control (CYP1A1) PCR product corresponded to 312 bp, and absence of the 480 bp and 215 bp bands indicated null genotypes for GSTM1 and GSTT1, respectively (Fig 1).

A total of 53 (21.9%) individuals had the GSTM1 deletion and among them, 25 were on ART, 17 were ART-naïve and 11 were healthy controls (Table 3). Among those with the GSTT1 deletion, 20 were on ART, 16 were ART-naïve and 12 were HIV seronegative controls (Table 3). The majority, 120 (49.6%), of the study population had both GSTM1 and GSTT1 present whilst 21 (8.7%) had both genes deleted (Table 3). The difference between the ART-naïve and control groups for the both null genotype was statistically significant (*p* < 0.05). There was however no significant difference for the other genotypes in the study groups (*p* > 0.05).

**Table 3:** GST genotypes distribution in study population.

Genotypes	ART (N = 105)	ART-naïve (N = 77)	Control (N = 60)	Total
GSTM1 null	25 (47.1)	17 (32.1)	11 (20.8)	53
GSTT1 null	20 (41.7)	16 (33.3)	12 (25.0)	48
GSTM1 + GSTT1	51 (42.5)	42 (35.0)	27 (22.5)	120
Both null	9 (42.9)	2 (9.2)	10 (47.6)	21

N is the population of the subgroups. Genotype frequency is expressed as n (%), where n is the total number of patients with defined genotype.

HIV patients with the GSTM1 deletion were 42, and 9 of them had CD4^+^ counts below 200 cells/mm^3^, 6 had CD4^+^ count between 200 cells/mm^3^ and 349 cells/mm^3^ whiles 27 people had their CD4^+^ count above 350 cells/mm^3^. Those with the GSTT1 deletion totalled 36. Among the HIV patients with both genes present, 23 had CD4^+^ count below 200, 15 had CD4^+^ count between 200 and 350 and 55 had CD4^+^ count more than 350. Persons with the GSTM1 or GSTT1 deletions were not associated with increased risk of having CD4^+^ count less than 350, but individuals who had both genes deleted were more likely to have CD4^+^ count above 350 cells/mm^3^ (Table 4).

**Table 4:** Association of GST genotypes with CD4^+^ count above 350 cells/mm^3^.

Genotypes	CD4^+^ count (cells/mm^3^)			Odds Ratio	95% CI	*p*-value
	>350	200–349	<200			
GSTM1 null	27	6	9	1.10	0.54–2.25	0.475
GSTT1 null	22	9	5	0.92	0.44–1.95	0.488
GSTM1+GSTT1	55	15	23	0.74	0.40–1.35	0.199
Both null	10	1	0	6.44	0.81–51.49	0.039*

*Statistically significant p-value.

The odds ratio, confidence intervals and p-values were obtained by a one-tail test analysis using the online tool from http://vassarstats.net/odds2x2.html. The comparison was done between patients with CD4^+^ counts >350 cells/mm3 and <350 cells/mm^3^.

**Fig 2:**
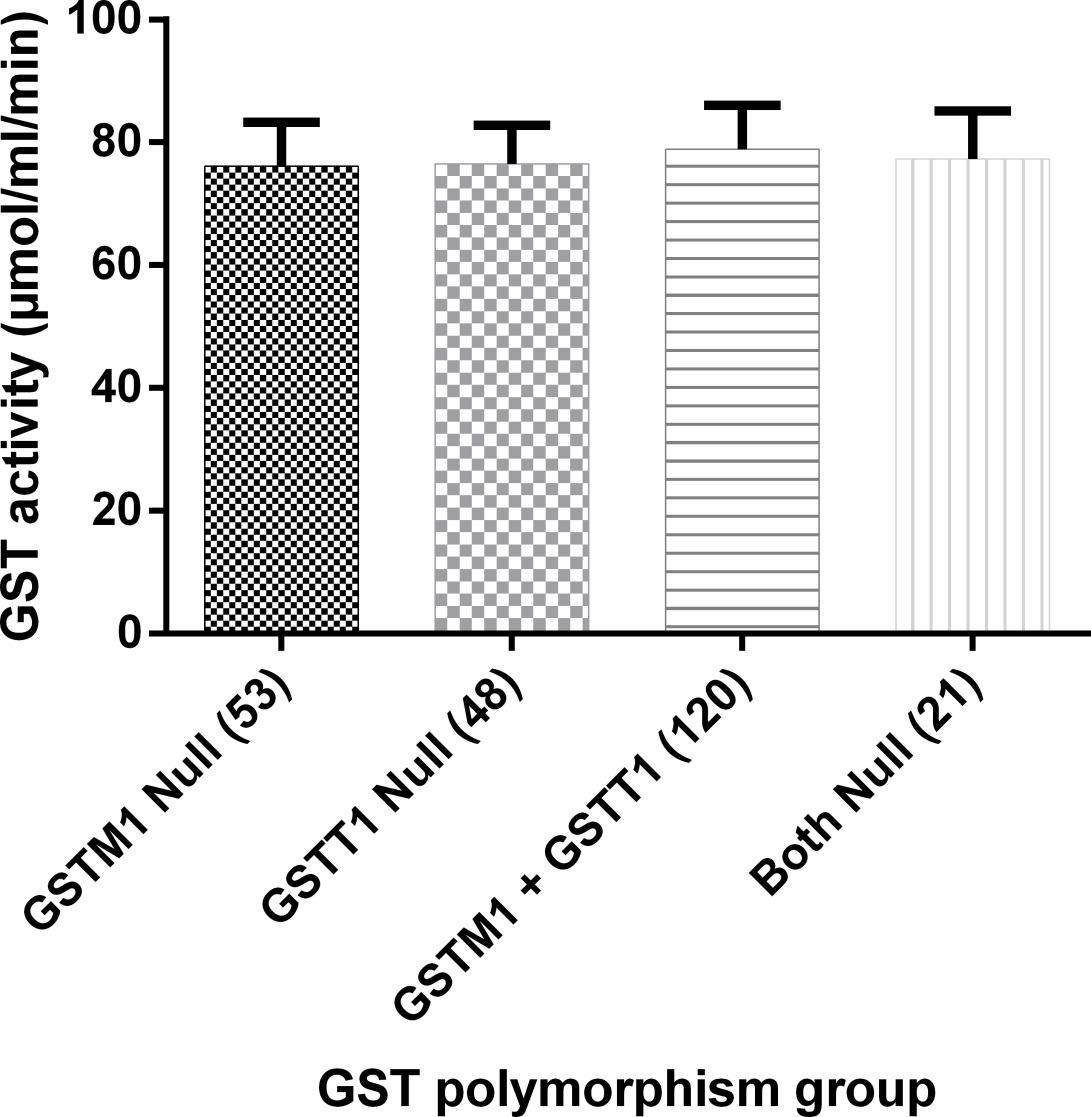
Effect of GST polymorphism on GST enzyme activity. GST polymorphism was obtained by PCR amplification of the GSTM1 and GSTT1 genes, with CYP1A1 gene used as control. GST enzyme activity was assayed spectrophotometrically.

GST activity in individuals that had both GSTM1 and GSTT1 present (79.35 ± 6.80) was not statistically different from the activity in those with either GSTM1 or GSTT1 absent, or with the both null genotype, suggesting that GST polymorphism does not affect GST enzyme activity (Fig 2).

## Discussion

The study, to the best of our knowledge is the first to assess the association between homozygous deletion of GSTM1 and GSTT1 genes with HIV/AIDS disease progression in Ghanaian patients. Higher average CD4^+^ counts were observed in patients on ART compared to their ART-naïve counterparts; making the naïve patients to be at increased risk of having opportunistic infections. The low CD4^+^ count in the individuals who are not on ART suggests a weaker immune system than the ART group and consistent with the ART-naïve group being at significant risk of acquiring opportunistic illnesses [22, 23]. ART is administered in order to mitigate HIV replication, which would subsequently lead to a rise in CD4^+^ count levels while providing protection against opportunistic infections [24, 25]. The protection provided by the therapy explains the generally high CD4^+^ count in the patients on ART in this study, although few individuals on ART also had CD4^+^ counts below 200 cells/mm^3^ (Table 2). Various studies have suggested that the CD4^+^ counts vary between people due to diverse factors such as recent vaccinations, exercise, lack of sleep, time of day, or smoking among others [26–28]. It is therefore possible that these factors could have contributed to the low CD4^+^ counts observed in some of the patients who are on ART, suggesting that a one-time CD4^+^ count may not be reliable and therefore recommended that the test be repeated once every three to six months [29].

In keeping with the relatively high CD4^+^ count, the viral load in the patients on ART was significantly lower than in the ART-naïve group. Viral load is a measure of plasma HIV RNA and reflects the levels of viral replication in the patients, and therefore the low viral load that was observed in the ART group in this study suggests that there was reduced risk of disease progression, and the high viral load in the ART-naïve patients suggests that viral replication was high and the infection could progress rapidly to AIDS [24].

In this present study, it was observed for the first time in Ghana that the frequency of deletion of GSTM1 was 21.9% whilst the GSTT1 was 19.8% (Fig 2). Parsons *et al*. in 2013 observed that the GSTM1 genotype that codes for a functional enzyme was not equally distributed by race [30], and different frequencies of the genes have been shown among three ethnic groups in Nigeria [4]. The functional GSTM1 enzyme has been shown to play a role in cancer prevention [31] and suggesting that the low frequency of GSTM1 gene deletion in Ghanaians in general will result in a better protection from the effects of reactive metabolites such as, benzo(a)pyrene, known to cause lung cancer than in Asians and Caucasian Whites [4]. Some pathways involving GST enzymes have however been shown to produce toxic products, and as a result, expression of GSTM1 and GSTT1, and exposure to certain compounds might pose a risk rather than confer protection [4, 32]. For instance, when methylchloride is exposed to GST, it is converted to formaldehyde which is more toxic [33]. The deleterious effects as well as the advantages of GST polymorphisms could be the reason why nature preserves the null phenotypes in populations.

Furthermore, when the GST polymorphisms were grouped by CDC staging of CD4^+^ count (Table 3), it was observed that HIV patients who had both GSTM1 and GSTT1 deleted were more likely to have CD4^+^ counts above 350 cells/mm^3^. This showed a weak but significant association between carriers of the GSTM1 and GSTT1 homozygous deletion and a favourable immune function. Moreover, this indicates that full loss of both GSTT1 and GSTM1 activities did not pose a risk of decreasing CD4^+^ count. It was expected that individuals with both genes deleted will not have the complete functional activity of the GST enzymes, and therefore respond poorly to drug metabolism with an associated low CD4^+^ count as previously reported [34]. Contrarily, the current results which showed no change in enzyme activity and elevated CD4^+^ count were not in agreement with the previous findings. This suggests that there could be a compensatory mechanism by other members of the GST family or other enzymes that play a role in metabolising xenobiotics. This was confirmed by the lack of significant difference in GST activity among the GST polymorphism groups (Fig 2). Studies by Gronau *et al*., 2003 and Konig-Greger *et al*., 2004 observed that persons with GSTM1 null mutation recorded similar GST enzyme activity as those with the functional GSTM1 enzyme [17, 18]. Furthermore, Bhattacharjee *et al*., 2013 used *in silico*, *in vitro* and *in vivo* approaches to prove that the absence of GSTM1 was compensated by the overexpression of GSTM2 [35].

Females were 64.9% of the study population; accounting for 57% of those on ART, 72.7% ART-naïve and 68.3% of the seronegative controls. Due to the high proportion of female to male HIV patients, some studies have suggested an increased risk in females getting infected. According to the CDC HIV Surveillance Report of 2014, the risk of a woman contracting HIV during intercourse is higher than it is for the man [19]. Besides, some of the women may not be aware of their male sexual partner’s risk factors for HIV; which may include multiple sexual partners, and drug abuse. Moreover, HIV policies in many countries have mandated pregnant women to undergo an HIV test and this could account for the reason why more women have tested positive than males. The World Health Organization (WHO) AIDS Epidemic Report of 2004 stated that sub-Saharan African women were more severely affected by HIV/AIDS, and those of reproductive age constituted about 57% of adults living with HIV, and this age accounted for up to 80% of HIV infected women in the world [36, 37].

The significant difference in age between patients on ART and those who are ART-naïve also backs the notion that HIV/AIDS infection may be acquired at an early age; possibly during the reproductive years of life. The strict eligibility criteria that qualifies an HIV patient to initiate ART (WHO recommends CD4^+^ <350 cells/mm^3^) could also be a contributing factor to the significant differences in age between those on ART and the ART-naïve group. This means that there is a time lag between the time some individuals test positive for HIV and the time ART is initiated.

## Conclusion

The findings of this study show that double deletion of glutathione S-transferases M1 and T1 with normal CD4^+^ count in patients diagnosed with HIV/AIDS may be as a result of compensatory effect to reduce oxidative stress.

## References

[1] HIV/AIDS JUNPo, HIV/AIDS JUNPo. Global AIDS update 2016. Geneva, Switzerland. 2016.

[2] Dabis F, Ekpini ER. HIV-1/AIDS and maternal and child health in Africa. Lancet (London, England). 2002;359(9323):2097–104. 10.1016/S0140-6736(02)08909-2 12086778

[3] WRW G., Rotstein OD, Jimenez M, Parodo J, Marshal JC. Augmented Intracellular Glutathione Inhibits Fas-Triggered Apoptosis of Activated Human Neutrophils. Blood. 1997;89:4175–81. 9166861

[4] Ebeshi BU, Bolaji OO, Masimirembwa CM. Glutathione-S-transferase (M1 and T1) polymorphisms in Nigerian populations. Journal of Medical Genetics and Genomics. 2011;3(4):56–60.

[5] Zhao L, Cox AG, Ruzicka JA, Bhat AA, Zhang W, Taylor EW. Molecular modeling and in vitro activity of an HIV-1-encoded glutathione peroxidase. Proc Natl Acad Sci U S A. 2000;97(12):6356–61. 10841544 10.1073/pnas.97.12.6356 PMC18607

[6] Strange JDHRC. Glutathione S-Transferase Polymorphisms and Their Biological Consequences. Pharmacology. 2000;61:154–66. doi: 10.1159/000028396 10971201 10.1159/000028396

[7] Soto-Quintana O, Zúñiga-González G, Ramírez-Patiño R, Ramos-Silva A, Figuera L, Carrillo-Moreno D, et al Association of the GSTM1 null polymorphism with breast cancer in a Mexican population. Genetics and Molecular Research. 2015;14(4):13066–75. doi: 10.4238/2015.October.26.2 26535619 10.4238/2015.October.26.2

[8] Pemble S, Schroeder K, Spencer S, Meyer D, Hallier E, Bolt H, et al Human glutathione S-transferase theta (GSTT1): cDNA cloning and the characterization of a genetic polymorphism. Biochemical Journal. 1994;300(1):271–6. 8198545 10.1042/bj3000271 PMC1138152

[9] DeMarini DM, Shelton ML, Warren SH, Ross TM, Shim JY, Richard AM, et al Glutathione S‐transferase‐mediated induction of GC→ AT transitions by halomethanes in salmonella. Environmental and molecular mutagenesis. 1997;30(4):440–7. 9435885

[10] Khabaz M, Nedjadi T, Gari M, Al-Maghrabi J, Atta H, Bakarman M, et al GSTM1 gene polymorphism and the risk of colorectal cancer in a Saudi Arabian population. Genet Mol Res. 2016;15:1. 10.4238/gmr.15017551 26909940

[11] Garte S, Gaspari L, Alexandrie A-K, Ambrosone C, Autrup H, Autrup JL, et al Metabolic gene polymorphism frequencies in control populations. Cancer Epidemiology and Prevention Biomarkers. 2001;10(12):1239–48. 11751440

[12] Adams CH, Werely CJ, C Victor T, Hoal EG, Rossouw G, Helden PDv. Allele frequencies for glutathione S-transferase and N-acetyltransferase 2 differ in African population groups and may be associated with oesophageal cancer or tuberculosis incidence. Clinical chemistry and laboratory medicine. 2003;41(4):600–5. doi: 10.1515/CCLM.2003.090 12747608 10.1515/CCLM.2003.090

[13] Moulik NR, Parveen F, Kumar A, Agrawal S. Glutathione-S-transferase polymorphism and acute lymphoblastic leukemia (ALL) in north Indian children: a case–control study and meta-analysis. Journal of human genetics. 2014;59(9):529–35. doi: 10.1038/jhg.2014.66 25102096 10.1038/jhg.2014.66

[14] Mokhtar GM, Sherif EM, Habeeb NM, Abdelmaksoud AA, El-Ghoroury EA, Ibrahim AS, et al Glutathione S-transferase gene polymorphism: Relation to cardiac iron overload in Egyptian patients with Beta Thalassemia Major. Hematology. 2016;21(1):46–53. doi: 10.1179/1607845415Y.0000000046 26288192 10.1179/1607845415Y.0000000046

[15] Wang J, Wang T, Yin G, Yang L, Wang Z, Bu X. Glutathione S-transferase polymorphisms influence chemotherapy response and treatment outcome in breast cancer. Genet Mol Res. 2015;14(3):11126–32. doi: 10.4238/2015.September.22.6 26400343 10.4238/2015.September.22.6

[16] Sun F-C, Jeng Y-C, Lee M-S, Wen C-F, Chen T-M, Lee M-S. Glutathione-S-Transferase M1 and T1 Gene Polymorphisms and Susceptibility to the Progression of Liver Fibrosis in Hcv-Infected Patients in Taiwan. Journal of Medical Biochemistry. 2014;33(3). doi: 10.2478/jomb-2013-0028

[17] Gronau S, Koenig-Greger D, Jerg M, Riechelmann H. Gene polymorphisms in detoxification enzymes as susceptibility factor for head and neck cancer? Otolaryngology-Head and Neck Surgery. 2003;128(5):674–80. doi: 10.1016/S0194-59980300176-1 12748560 10.1016/S0194-59980300176-1

[18] Konig-Greger D, Riechelmann H, Wittich U, Gronau S. Genotype and phenotype of glutathione-S-transferase in patients with head and neck carcinoma. Otolaryngology—Head and Neck Surgery. 2004;130(6):718–25. doi: 10.1016/j.otohns.2003.10.011 15195058 10.1016/j.otohns.2003.10.011

[19] CDC. Diagnoses of HIV infection in the United States and dependent areas. HIV Surveillance Report, Vol. 23. 2013.

[20] Gattás GJF, Universidade de São P, Kato M, Fundacentro-Crba SB, University of North Carolina CHUSA, Soares-Vieira JA, et al Ethnicity and glutathione S-transferase (GSTM1/GSTT1) polymorphisms in a Brazilian population. Braz J Med Biol Res. 2004;37(4):451–8. 15064808 10.1590/s0100-879x2004000400002

[21] Chikezie P, Chikezie C, Uwakwe A, Monago C. Comparative Study of Glutathione S-Transferase Activity of Three Human Erythrocyte Genotypes Infected With Plasmodium falciparum. Journal of Applied Sciences and Environmental Management. 2009;13(3).

[22] Merson MH, O'Malley J, Serwadda D, Apisuk C. The history and challenge of HIV prevention. Lancet (London, England). 2008;372(9637):475–88. 10.1016/S0140-6736(08)60884-3 18687461

[23] Quaye IK, Brandful J, Ekuban FA, Gyan B, Ankrah NA. Haptoglobin polymorphism in human immunodeficiency virus infection: Hp0 phenotype limits depletion of CD4 cell counts in HIV-1-seropositive individuals. The Journal of infectious diseases. 2000;181(4):1483–5. doi: 10.1086/315377 10762581 10.1086/315377

[24] Costagliola D, Lacombe JM, Ghosn J, Delaugerre C, Pialoux G, Cuzin L, et al CD4+ cell count recovery in naive patients initiating cART, who achieved and maintained plasma HIV-RNA suppression. Journal of the International AIDS Society. 2014;17(4 Suppl 3):19481. 25393990 10.7448/IAS.17.4.19481 PMC4224828

[25] Corbeau P, Reynes J. Immune reconstitution under antiretroviral therapy: the new challenge in HIV-1 infection. Blood. 2011;117(21):5582–90. doi: 10.1182/blood-2010-12-322453 21403129 10.1182/blood-2010-12-322453

[26] BØYum A, Norwegian Defence Research Establishment DfETK, Wiik P, Norwegian Defence Research Establishment DfETK, E Gustavsson, Nycomed Pharma O, et al The Effect of Strenuous Exercise, Calorie Deficiency and Sleep Deprivation on White Blood Cells, Plasma Immunoglobulins and Cytokines. Scandinavian Journal of Immunology. 2016;43(2):228–35. 10.1046/j.1365-3083.1996.d01-32.x 8633203

[27] Sullivan PS, Hanson DL, Dworkin MS, Jones JL, Ward JW. Effect of influenza vaccination on disease progression among HIV-infected persons. AIDS (London, England). 2000;14(17):2781–5. 10.1097/00002030-200012010-00018 11125897

[28] Nielsen HG, Øktedalen O, Opstad P-K, Lyberg T. Plasma Cytokine Profiles in Long-Term Strenuous Exercise. Journal of Sports Medicine. 2016;2016. 10.1155/2016/7186137 PMC4864530 27239554

[29] Gale HB, Gitterman SR, Hoffman HJ, Gordin FM, Benator DA, Labriola AM, et al Is frequent CD4+ T-lymphocyte count monitoring necessary for persons with counts≥ 300 cells/μL and HIV-1 suppression? Clinical Infectious Diseases. 2013;56(9):1340–3. doi: 10.1093/cid/cit004 23315315 10.1093/cid/cit004 PMC3693489

[30] Parsons M, Campa A, Lai S, Li Y, Martinez JD, Murillo J, et al Effect of GSTM1-polymorphism on disease progression and oxidative stress in HIV infection: modulation by HIV/HCV co-infection and alcohol consumption. Journal of AIDS & clinical research. 2013;4(9). 10.4172/2155-6113.1000237 PMC3887471 24416632

[31] Ntais C, Polycarpou A, Ioannidis JP. Association of GSTM1, GSTT1, and GSTP1 gene polymorphisms with the risk of prostate cancer: a meta-analysis. Cancer Epidemiology and Prevention Biomarkers. 2005;14(1):176–81. 15668493

[32] Barahmani N, Carpentieri S, Li X-N, Wang T, Cao Y, Howe L, et al Glutathione S-transferase M1 and T1 polymorphisms may predict adverse effects after therapy in children with medulloblastoma. Neuro-oncology. 2009;11(3):292–300. doi: 10.1215/15228517-2008-089 18952980 10.1215/15228517-2008-089 PMC2718973

[33] Josephy PD. Genetic variations in human glutathione transferase enzymes: significance for pharmacology and toxicology. Human genomics and proteomics. 2010;2(1). 10.4061/2010/876940 PMC2958679 20981235

[34] Singh HO, Lata S, Angadi M, Bapat S, Pawar J, Nema V, et al Impact of GSTM1, GSTT1 and GSTP1 gene polymorphism and risk of ARV-associated hepatotoxicity in HIV-infected individuals and its modulation. Pharmacogenomics J. 2017;17(1):53–60. doi: 10.1038/tpj.2015.88 26667829 10.1038/tpj.2015.88

[35] Bhattacharjee Pritha PS, Banerjee Mayukh, Patra Deblina, Banerjee Priyam, Ghoshal Nanda, Bandyopadhyay Arun & Giri Ashok K. Functional compensation of glutathione S-transferase M1 (GSTM1) null by another GST superfamily member, GSTM2. Nature Scientific Reports. 2013;3(2704). 10.1038/srep02704 PMC3776957 24048194

[36] Dabis F, Ekpini ER. HIV-1/AIDS and maternal and child health in Africa. The Lancet. 2002;359(9323):2097–104. 10.1016/S0140-6736(02)08909-2 12086778

[37] UNAIDS/WHO. AIDS epidemic update. UNAIDS/WHO, 2004 December 2004.

